# 

**Published:** 1833-07-01

**Authors:** 


### MEDICO-CHIRURGICAL REVIEW,

AND

### JOURNAL

OF

### PRACTICAL MEDICINE.

*(NEW SERIES.)*

VOLUME NINETEEN,

[1st of JULY to 30th of SEPTEMBER,]

1833.

VOL. XXIII. of ANALYTICAL SERIES.

nothing to index

EDITED BY JAMES JOHNSON, M.D.

PHYSICIAN EXTRAORDINARY TO THE KING,

*&c. &c. &c.*

Surgeon Genl's Office
LIBRARY
57454
Washington, D.C.

LONDON:

PUBLISHED BY S. HIGHLEY, 32, FLEET STREET,

AND WEBB STREET, ST. THOMAS'S HOSPITAL.

*Re-printed in New York, by Mr. Wood.*

1833.

W1
ME75T

PRINTED BY G. HAYDEN,

Little College Street, Westminster.

### IV. Medical Politics.

#### Apothecaries.

### BIBLIOGRAPHICAL RECORD;

OR,

*Works received for Review since the last Quarter.*

---

1. A Treatise on the Physiology and Diseases of the Eye; containing a new Mode of curing Cataract without Operation—Experiments and Observations on Vision, &c. together with Remarks on the Preservation of Sight, and on Spectacles, Reading-Glasses, &c. By John Harrison Curtis, Esq. Oculist, &c. Octavo, pp. 222, with a coloured Plate, price 7*s.* 6*d.* bds. Longman and Co. March, 1833.

☞ *There is more originality, as well as research, in this volume on the eye, than in Mr. Curtis's work on the Ear, although the latter has gone through five editions.*

---

2. Observations on Impediments of Speech; with some Remarks on their successful Treatment. In a Letter to T. J. Pettigrew, Esq. By Richd. Cull. 8vo. pp. 31. Renshaw and Rush, March, 1833.

☞ *There are some excellent hints in this little pamphlet, which are well worthy of the medical practitioner's attention.*

3. Hortus Medicus, or Figures and Descriptions of the more important Plants used in Medicine, or possessed of poisonous Qualities; with their Medical Properties, Chemical Analyses, &c. By GEORGE GRAVES, F.L.S, &c. and JOHN DAVIE MORRIES, M.D. Quarto, pp. 32, and 12 coloured Plates. No. I. 7*s*. 6*d*. plain, 10*s*. 6*d*. coloured. A. and C. Black, Edinburgh, March, 1833.

4. A plain Statement of Vaccination; designed for the Heads of Families, wherein the History, Advantages, and Errors of this Subject are popularly treated. By A. B. C. Duodecimo, pp. 58. Renshaw and Rush, 1833.

☞ *This little brochure is very well calculated to effect the purpose for which it was compiled.*

5. Principles of Pathology and Practice of Physic. By JOHN MACKINTOSH, M.D. Lecturer on the Practice of Physic in Edinburgh, &c. Two Volumes, 8vo, 3d Edition, 1833.

☞ *This third Edition has been considerably enlarged—every sentence has been reconsidered, and many errors have been corrected. It may now be considered as the most standard work of the kind in the English language.*

6. A Treatise on the Venereal Disease and its Varieties. By WILLIAM WALLACE, M.R.I.A. &c. Surgeon to the Jervis Street Infirmary, Dublin, &c. Octavo, pp. 382. Burgess and Hill, April, 1833, price 12*s*. bds.

7. Succinct Practical Observations on the Effects of Bloodletting, &c. By ED. GEO. GEOGHEGAN, M.R.C.S. I. Octavo, pp. 60. Longman's, April, 1833.

3. Illustrations of the Mechanism of Parturition. By CHARLES EMILIUS ROSS, A.M. M.B. Quarto, six Plates, lithographed, with numerous Figures.

☞ *These Plates, which are very cheap, will be found extremely useful to the obstetric student.*

9. The Principles and Practice of Obstetric Medicine. By D. D. DAVIS, M.D. &c. Part XIX. Taylor, London, May, 1833, price 2*s*.

10. An Address to the Governors of the Birmingham Hospital, on the Appointment of Assistant Surgeons to that Institution. By RICHARD MIDDLEMORE, M.R.C.S.

11. A Manual of Pharmacy. By WILLIAM THOMAS BRANDE, F.R.S. &c. Third Edition, corrected and enlarged. Octavo, pp. 544, price 14*s*. May, 1833.

☞ *This highly valuable Work is greatly enlarged and improved, so as to constitute a standard production. It may be considered, in fact, as the substance of the annual Course of Lectures delivered by the author at Apothecaries' Hall.*

12. Observationes quædam de Entero-Helcosi. Dissertatio Inauguralis Medica quam gratiosi Medicorum ordinis Auctoritate in Academia Lipsiensi Summorum in Medicina et Chirurgia Honorum rite, &c. Auctor GULIELMUS EDWARDUS SWAINE, Londinensis, Medicinæ Baccalaureus. Quarto, pp. 31, with a Lithographic Plate. Leipsic, 1832.

☞ *This is a very respectable thesis on ulceration of the intestines, as connected with fever, &c.*

13. Graphic Illustrations of Abortion, and the Diseases of Menstruation; consisting of twelve Plates, from Drawings engraved on Stone, and coloured by Mr. J. Perry, and two Copper Plates from the Philosophical Transactions, coloured by the same Artist; the whole representing forty-five Specimens of aborted Ova, and adventitious Productions of the Uterus, with preliminary Observations, Explanations of the Figures, and Remarks, Anatomical and Physiological. By A. B. GRANVILLE, M.D. F.R.S. &c. Physician-Accoucheur to the Westminster General Dispensary, &c. Quarto, pp. 51, with numerous coloured Plates, price, by Subscription, £1. 15*s*. June, 1833.

14. Observations on the Testicles. By JAMES RUSSELL, F.R.C.S. late Regius Professor of Clinical Surgery in the University of Edinburgh. Small 8vo, pp. 276. Longman and Co. May, 1833.

15. Hortus Medicus; or Figures and Descriptions of the more important Plants used in Medicine, or possessed of poisonous Qualities; with their Medical Properties, Chemical Analysis, &c. By George Graves, Fellow of the Linnæan Society, &c. and John Davie Morries, M.D. &c. No. II. price 7*s*. 6*d*. plain, 10*s*. 6*d*. coloured. Black, Edinburgh, May, 1833.

☞ *A very meritorious enterprize.*

---

16. An Inquiry into the Causes of Respiration, of the Motion of the Blood, Animal Heat, Absorption, and Muscular Motion; with Practical Inferences. By James Carson, M.D. of Liverpool. Octavo, pp. 447, with a Plate. Second Edition, greatly enlarged, 1833.

---

17. Synopsis of a Course of Lectures on the Practice of Medicine. By Chas. Lendrick, M.D. King's Professor, &c. Ireland.

---

18. Introductory Lecture, addressed to the Students in the School of Physic (Dublin). By Charles Lendrick, M.D. &c. Octavo, pp. 21. May, 1833.

☞ *A very sensible address.*

---

19. The Origin and Progress of the Malignant Cholera in Manchester. With an illustrative Chart. By Henry Gaulter, M.D. of Magdalen Hall, Oxford. Octavo, pp. 206, with Chart. June, 1833.

☞ *This is one of the most able pamphlets on cholera which we have seen. The author advocates, we may say* proves, *the local or spontaneous origin of cholera in every part of the globe, as well as in Manchester; while he admits the possibility of the disease being sometimes communicated from individual to individual.*

---

20. Principles and Practice of Obstetric Medicine, &c. By Dr. Davis. Part XX. with Plates. June, 1833.

---

21. Principles and Illustrations of Morbid Anatomy, adapted to the Elements of M. Andral's, and to the Cyclopædia of Practical Medicine, &c. By J. Hope, M.D. Parts IV. and V., price 8*s*. 6*d*. each.

☞ *These two Parts are occupied with diseases of the liver, and the plates support the reputation of the preceding fasciculi.*

---

22. Sketches from the Case-Book, to illustrate the Influence of the Mind on the Body, with the Treatment of some of the most important Brain and Nervous Disturbances which arise from this Influence. By R. Fletcher, Esq. Surgeon to the Gloucester General Hospital, &c. Octavo, pp. 391. June, 1833.

---

23. Some Observations on a Letter from Dr. N. Chapman, of Philadelphia, to Dr. W. B. Tyler, on the Subject of Cholera, as appearing in Philadelphia, August, 1832. Octavo, sewed, 1833.

---

24. Some Observations on the Subject of the Jalap Plant. By J. R. Coxe, M.D. Octavo, sewed, pp. 20.

---

25. Observations on the Illusions of the Insane, and on the Medico-legal Question of their Confinement. Translated from the French of M. Esquirol. By William Liddell, M.R.C.S. Octavo, pp. 89. Renshaw and Rush, June, 1833.

---

26. Instituicoes de Medicina Forense. Par Jose Ferreira Borges. Octavo, pp. 570. Paris 1832.

☞ *This appears to be an able compilation, in the Portuguese language, on the subject of forensic medicine. The author, we understand, resides in London.*

---

27. The Calamities of Genius illustrated, by referring the Anomalies in the Literary Character to the Habits and Constitutional Peculiarities of Men of Genius. By R. R. Madden, Esq. Author of Travels in Turkey, &c. Small 8vo, two Volumes, June, 1833.

☞ *This is a medical work, notwithstanding its title, and an extremely amusing one it is. It traces the influence of bodily disorder on the mind and temper with great ability, and illustrates it by appropriate examples. We shall review the work in our next.*

---

28. The Analysis of Inorganic Bodies. By J. J. Berzelius. Translated from the French Edition, by G. O. Rees. Small Octavo, pp. 164. London, June, 1833.

---

29. A Treatise on some Nervous Disorders: being chiefly intended to illustrate those varieties which simulate Structural Disease. By Edward Lee, M.R.C.S. and formerly House-Surgeon of St. George's Hospital. 8vo. pp. 152. Burgess and Hill, June, 1833.

